# Partial-thickness macular hole in vitreomacular traction syndrome: a case report and review of the literature

**DOI:** 10.1186/1752-1947-4-7

**Published:** 2010-01-13

**Authors:** Niranjan Kumar, Jamal Al Kandari, Khalid Al Sabti, Vivek B Wani

**Affiliations:** 1 Department of Ophthalmology, Al Bahar Ophthalmology Center, Ibn Sina Hospital, Kuwait City, Kuwait

## Abstract

**Introduction:**

Vitreomacular traction syndrome has recently been recognized as a distinct clinical condition. It may lead to many complications, such as cystoid macular edema, macular pucker formation, tractional macular detachment, and full-thickness macular hole formation.

**Case presentation:**

We report a case of vitreomacular traction syndrome with eccentric traction at the macula and a partial-thickness macular hole in a 63-year-old Pakistani Punjabi man. The patient was evaluated using optical coherence tomography, and he underwent a successful pars plana vitrectomy. After the operation, his foveal contour regained normal configuration, and his visual acuity improved from 20/60 to 20/30.

**Conclusions:**

Pars plana vitrectomy prevents the progression of a partial thickness macular hole in vitreomacular traction syndrome. The relief of traction by vitrectomy restores foveal anatomy and visual acuity in this condition.

## Introduction

Vitreomacular traction syndrome results in many complications, such as cystoid macular edema, macular pucker formation, tractional macular detachment, retinal blood vessel avulsion, and macular hole formation [1,2]. In a minority of reported cases, it resolves spontaneously due to complete posterior vitreous detachment [3]. However, the development of a partial-thickness macular hole in vitreomacular traction syndrome and its surgical outcome is not well described in the literature. We report a case of vitreomacular traction syndrome complicated by the development of a partial-thickness macular hole. The condition was treated successfully using pars plana vitrectomy.

## Case presentation

**Figure 1:**
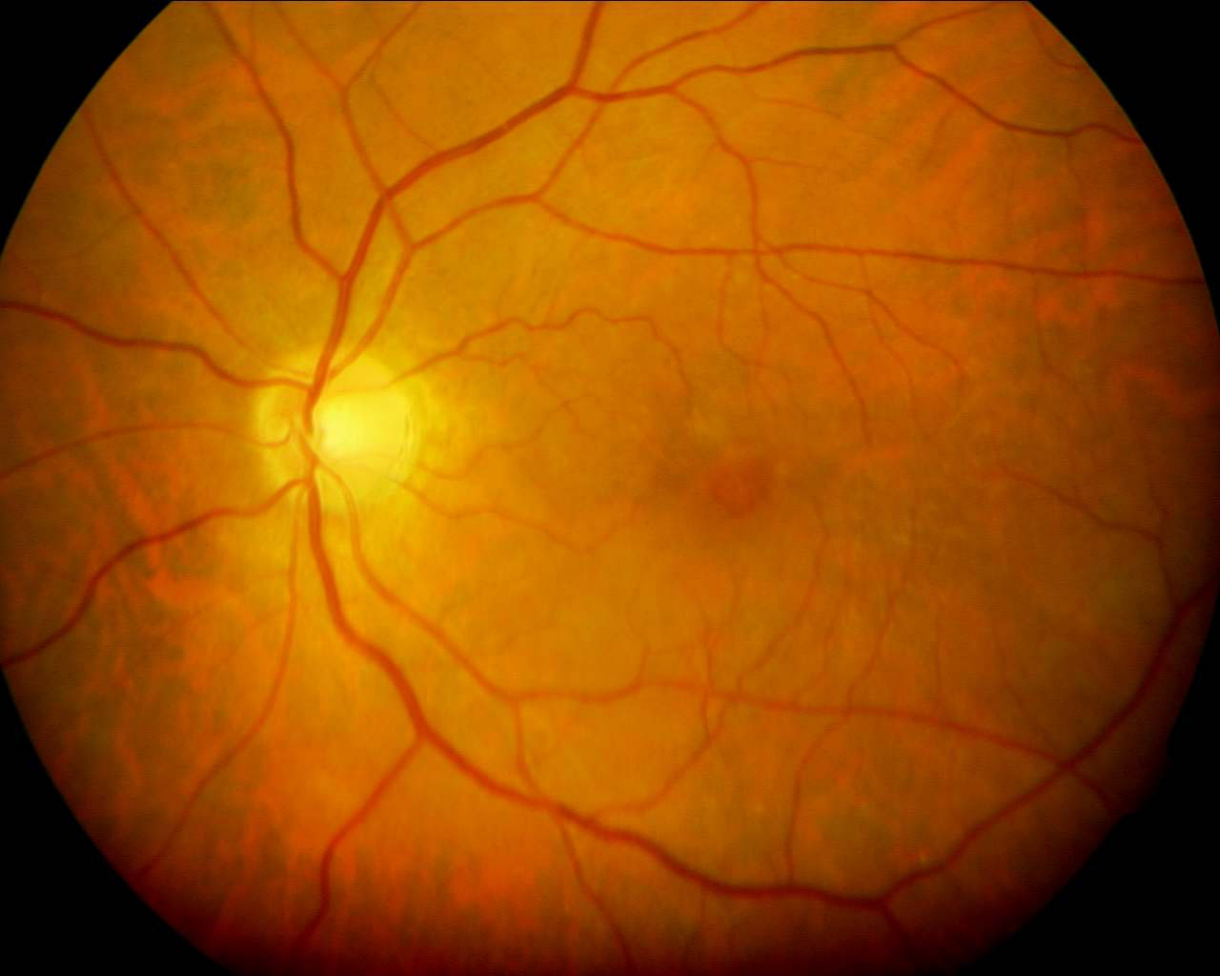
**This preoperative fundus photograph shows a partial-thickness macular hole**.

A 63-year-old Pakistani Punjabi man presented to our hospital with gradual diminution of vision in his left eye for the last six months. He had diabetes and was on oral hypoglycemic agents for the last four years. He did not have a history of refractive error, ocular inflammation, or surgery. On examination, his best corrected visual acuity was 20/20 in his right eye and 20/60 in his left eye. Anterior segment examination was unremarkable except for the finding that he had mild cortical lens changes in both eyes. Fundus examination by slit lamp biomicroscopy showed that he had an epiretinal membrane at the macula in his right eye and a vitreomacular traction causing a partial-thickness macular hole in his left eye (Figure 1). The traction was seen superior and temporal to the macula. There was no evidence of diabetic retinopathy in either eye.

**Figure 2:**
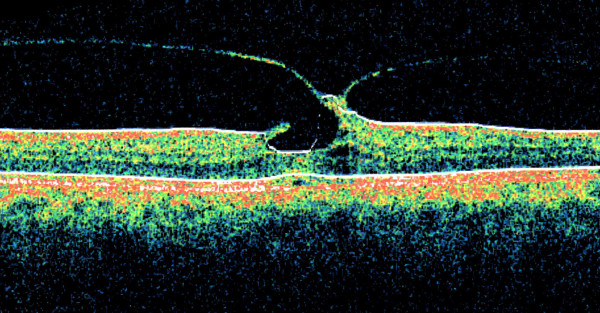
**This preoperative optical coherence tomography image shows the presence of eccentric vitreomacular traction, a partial-thickness macular hole, intraretinal cysts, and a small retinal pigment epithelial detachment**.

**Figure 3:**
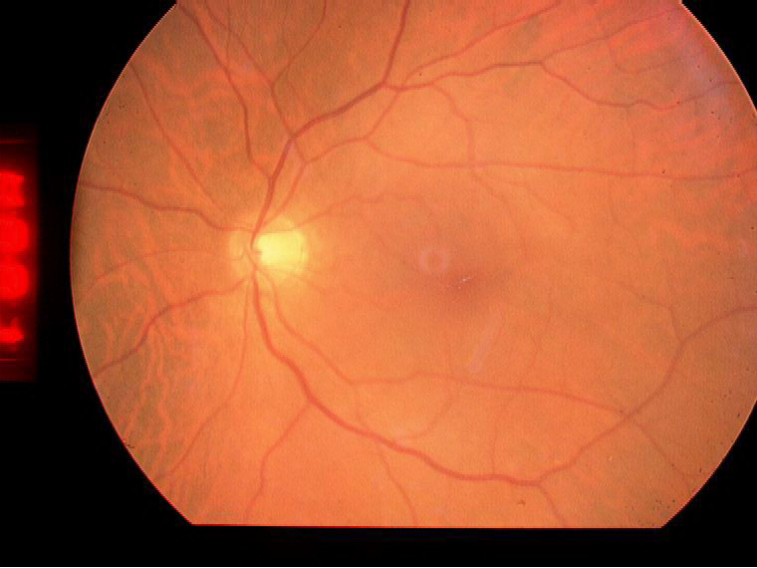
**This postoperative fundus photograph shows that the macular hole has been closed**.

Our patient underwent fluorescein angiography and optical coherence tomography (OCT) (Stratus OCT™: Carl Zeiss Meditec, Dublin, California). The OCT examination of his left eye confirmed the clinically noted findings and showed that his left eye had thick vitreomacular traction, intraretinal cysts, and small retinal pigment epithelial (RPE) detachment (Figure 2). We performed a pars plana vitrectomy (PPV), removal of the posterior hyaloid, a fluid-air exchange, and an 18% sulfur hexafluoride (SF_6_) gas injection. He maintained strict postoperative prone positioning for one week. After the operation, the hole closed clinically (Figure 3).

**Figure 4:**
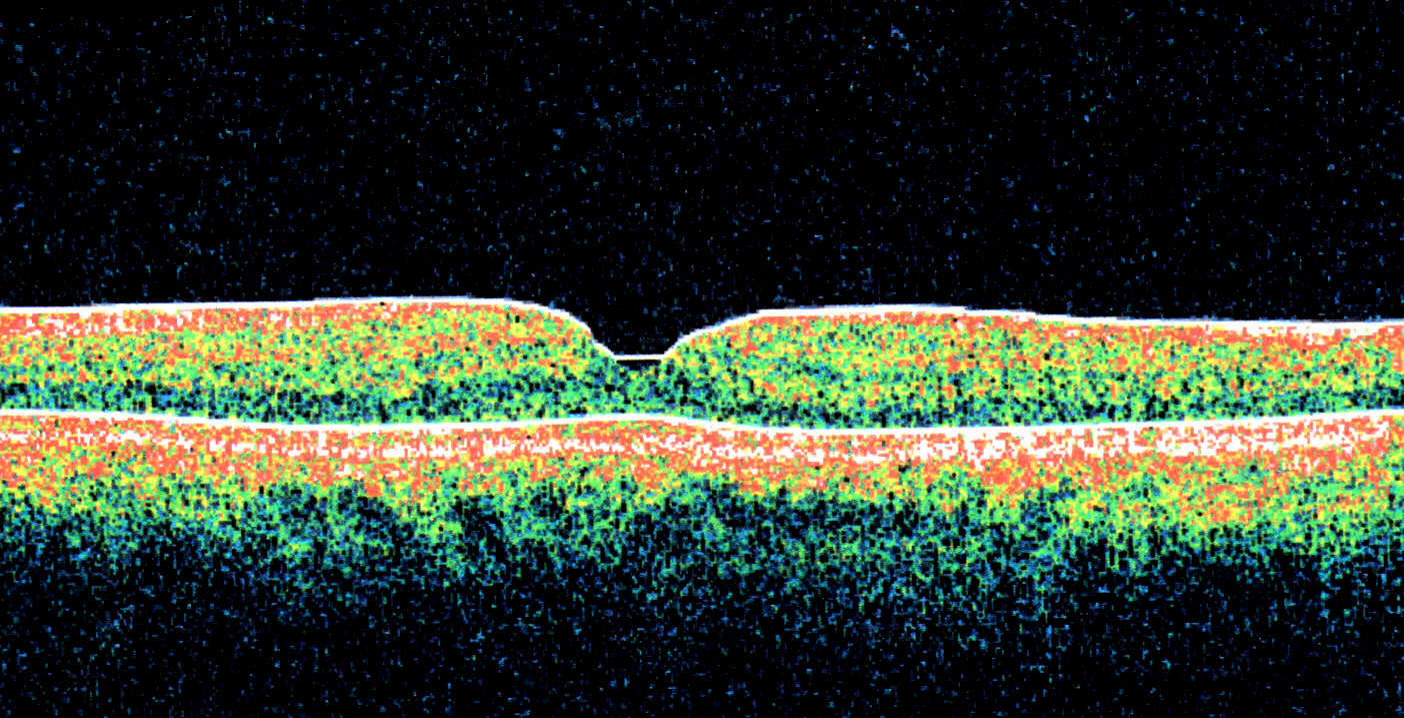
**This postoperative optical coherence tomography image shows the absence of a partial-thickness macular hole, intraretinal cysts, and retinal pigment epithelial detachment**.

Three months after the operation, his OCT showed the absence of the partial-thickness macular hole and resolution of the intraretinal cysts and RPE detachment (Figure 4). He did not develop potential complications like the development of a full-thickness macular hole, progression of a cataract, retinal detachment, or endophthalmitis. His visual acuity improved to 20/40 by the third postoperative month, and finally achieved 20/30 by the sixth month. He has maintained this level of visual acuity and a flat macula for the past 18 months.

## Discussion

Vitreomacular traction syndrome is caused by partial posterior vitreous detachment. The posterior hyaloid face remains attached to the macula and causes anterior-posterior traction. This traction usually results in anatomical and functional changes in the macula [1,2]. Although complete posterior vitreous detachment may result in the resolution of the condition, such a favorable outcome is uncommon [3]. Macular changes described in vitreomacular traction syndrome include cystic changes, macular pucker formation, macular detachment, and full-thickness macular hole formation [1]. The etiology of vitreomacular syndrome includes diabetic retinopathy, myopia, inflammation of the eye, and idiopathic disease.

We report here the development of a partial-thickness macular hole due to vitreomacular traction syndrome and its surgical management. This complication of vitreomacular traction syndrome and its successful management by vitreous surgery is not well described in the literature. This condition is difficult to diagnose by slit lamp biomicroscopy. Scanning laser ophthalmoscopy and OCT examination have recently been used in the diagnosis and follow-up of vitreomacular traction syndrome [4-6]. An OCT examination showed a definite eccentric vitreomacular traction and partial-thickness macular hole in our patient. Additionally, intraretinal cysts and RPE detachment were observed on OCT examination.

Hashimoto *et al. *reported a case of macular detachment caused by vitreomacular traction [2]. They found thick adhesions covering the detached macula. On the other hand, our patient had a localized adhesion, which might have prevented the development of macular detachment. Figus *et al. *[4] demonstrated incomplete posterior vitreoschisis in a case of vitreomacular syndrome with an impending macular hole. Giacomo and Andrea [5] reported a lamellar hole in myopic traction maculopathy. Our patient had idiopathic vitreomacular traction syndrome. Yamada and Kishi [6] described two anatomical types of vitreomacular traction. In their series, one group of patients had V-shaped traction that was centered on the macula, while the other group had eccentric, nasally-attached vitreomacular traction. Our patient had eccentric traction on the macula that was localized superiorly and temporally.

We performed PPV with the removal of the posterior hyaloid, fluid-air exchange, and SF_6 _gas injection. Internal limiting membrane peeling was not performed to avoid possible formation of a full-thickness macular hole. PPV in the management of vitreomacular traction syndrome has been described by others [6-10]. Yamada and Kishi [6] achieved good surgical results with normal foveal configuration after performing PPV in their patients with V-shaped attachment. However, in patients with eccentric vitreomacular traction, a macular hole developed in two of their patients, while a persistent macular edema developed in one patient. We achieved normal foveal configuration without these complications in our patient.

Smiddy *et al. *[7] were able to release traction in all of their patients without complications. However, they did not describe OCT findings in their patients. McDonald *et al. *[8] reported the results of PPV in 20 patients. They described "classic" and "variable" types of vitreomacular syndrome. Those considered classic had 360-degree mid-peripheral vitreous detachment, while the variable type had a variety of mid-peripheral vitreous separation. However, they did not describe types of attachment at the macula.

Sonmez *et al. *[9] described three types of anatomical configuration in a series of 24 patients. They performed PPV in all these patients. Group 1 had focal vitreofoveal hyaloidal attachment with perifoveal separation. Group 2 had vitreoretinal hyaloidal attachment to the macula and papillomacular bundle. Group 3 had broad vitreofoveal attachment with fine epiretinal membrane over the posterior pole. They achieved a better outcome in Group 1 cases. Our patient had localized eccentric traction with a lamellar macular hole, intraretinal cysts, and RPE detachment.

Georgalas *et al. *[10] reported a case of vitreomacular traction with retinal pigment epithelial detachment. There was limited improvement in the visual acuity of their patient after they performed PPV with internal limiting membrane peeling. RPE detachment persisted for 11 months in their patient, while it resolved within 3 months in ours.

It is difficult to recommend the appropriate timing or indications of surgical intervention in the patients described above. As such, we decided in favor of surgical intervention due to the progressive diminution of our patient's vision, which reached 20/60, and OCT findings like the presence of intraretinal cysts and retinal pigment epithelial detachment in addition to thick eccentric traction. Underlying retinal conditions like myopia, diabetic retinopathy and ocular inflammation that cause irreversible damage to the fovea may limit a patient's visual recovery after PPV for a partial-thickness macular hole with vitreomacular traction. This should also to be taken into consideration when planning surgical intervention in cases of vitreomacular traction syndrome.

## Conclusions

We reported a case of a partial-thickness macular hole with eccentric attachment at the macula, documented with OCT and successfully treated by PPV. OCT showed the precise attachment at the macula. PPV and the removal of posterior hyaloid prevents traction and further damage to the macula and restores the normal macular configuration with improvement in the visual acuity. However, a randomized case-control study is needed to identify the future course of the disease and its long-term surgical outcome.

## Abbreviations

OCT: optical coherence tomography; PPV: pars plana vitrectomy; RPE: retinal pigment epithelial.

## Consent

Written informed consent was obtained from the patient for publication of this case report and any accompanying images. A copy of the written consent is available for review by the Editor-in-Chief of this journal.

## Competing interests

The authors declare that they have no competing interests.

## Authors' contributions

NK diagnosed the patient, performed the surgery, and designed the case report. JK, KS and VB contributed to the writing of the manuscript, carried out the literature research, and performed a critical analysis of the manuscript. All authors read and approved the final manuscript.
